# Spatiotemporal information enhanced multi-feature short-term traffic flow prediction

**DOI:** 10.1371/journal.pone.0306892

**Published:** 2024-07-15

**Authors:** Deqi Huang, Jiajia He, Yating Tu, Zikuang Ye, Lirong Xie

**Affiliations:** College of Electrical Engineering, Xinjiang University, Ürümqi, China; National Institute of Technology Rourkela, INDIA

## Abstract

Accurately predicting traffic flow is crucial for optimizing traffic conditions, reducing congestion, and improving travel efficiency. To explore spatiotemporal characteristics of traffic flow in depth, this study proposes the MFSTBiSGAT model. The MFSTBiSGAT model leverages graph attention networks to extract dynamic spatial features from complex road networks, and utilizes bidirectional long short-term memory networks to capture temporal correlations from both past and future time perspectives. Additionally, spatial and temporal information enhancement layers are employed to comprehensively capture traffic flow patterns. The model aims to directly extract original temporal features from traffic flow data, and utilizes the Spearman function to extract hidden spatial matrices of road networks for deeper insights into spatiotemporal characteristics. Historical traffic speed and lane occupancy data are integrated into the prediction model to reduce forecasting errors and enhance robustness. Experimental results on two real-world traffic datasets demonstrate that MFSTBiSGAT successfully extracts and captures spatiotemporal correlations in traffic networks, significantly improving prediction accuracy.

## Introduction

With the improvement of China’s economic level, more and more families choose to travel by private car, which not only increases the chance of traffic congestion, but also brings serious challenges to traffic management [1]. Traffic flow prediction can assist citizens in planning their travel routes in advance, avoiding congested sections and saving fuel costs. For traffic management departments, the prediction results can help them formulate diversion and control strategies, reduce the probability of accidents, and ultimately achieve the goals of efficient resource utilization, reduced energy consumption, environmental protection, and alleviating travel pressure [2]. The development of traffic flow prediction algorithms is mainly divided into three directions: statistical analysis theory, machine learning and deep learning.

Statistical theory analysis methods used for traffic flow prediction include Kalman filters (KF) [3], historical average method (HA) [4], autoregressive integrated moving average model (ARIMA) [5], etc. However, these models are sensitive to noise and the selection of model parameters, and they are difficult to handle the nonlinear relationships in complex data, resulting in unsatisfactory performance. Therefore, some scholars have attempted to use machine learning methods for traffic flow prediction, such as artificial neural networks (ANN) [6], k-nearest neighbors (KNN) [7] and support vector regression (SVR) [8], among other methods. Cui et al. applied backpropagation neural network (BP) to improve the training effect and generalization ability of the network by training the network with small error and using its weight vector as the initial value for the next network, together with adaptive learning rate and additional momentum method [9]. Zhang et al. [10] used a BPNN model to predict the traffic flow during peak hours on a non-topological road segment in Beijing. These machine learning models are typically capable of handling complex data and nonlinear relationships. However, they have higher requirements for data volume and feature engineering, and there is a risk of overfitting.

With the deepening research on deep learning, researchers have begun to explore the use of deep learning [11] methods for traffic flow prediction. In the field of traffic flow prediction, commonly used deep learning models include Convolutional Neural Networks (CNN) [12], Recurrent Neural Networks (RNN) [13, 14], Graph Neural Networks (GNN) [15, 16] and so on. For example, Yao et al. proposed a traffic prediction method that combines CNN and Long Short-Term Memory (LSTM) to jointly model spatial and temporal dependencies [17]. Xu et al. [18] introduced a new model called C-BiLSTM, which combines Convolutional Neural Networks with Bidirectional Long Short-Term Memory Networks. This model extracts local spatial features and forward-backward temporal features to better capture the spatio-temporal characteristics of traffic flow. Although CNN can automatically learn high-level features from data [19], it needs to consider the network structure selection problem when extracting spatial features of roads, and the size and number of grids need to be set manually, which has some limitations for extracting spatial features of road topology.

Compared to CNN, Graph Convolutional Networks (GCN) have better adaptability and accuracy in extracting spatial features of roads. The main reason is that GCN can leverage the road network’s topological structure to perform convolutions on nodes and edges, thus extracting road spatial features. Moreover, the convolutional kernels in GCN can adaptively adjust, significantly improving the model’s robustness. Based on these advantages, numerous researchers have conducted studies on traffic flow prediction by incorporating GCN. For instance, Yu et al. [20] constructed a spatio-temporal convolution block similar to a “sandwich” structure by combining graph convolution and gated temporal convolution in order to fuse spatio-temporal and spatial features, which can extract useful spatial features and basic temporal features. Guo et al. [21] proposed the ASTGCN model, which employs a stacked model structure, combining multiple STGCNs and an attention mechanism to form a multilayered structure, in order to further improve the model’s accuracy and robustness. Fang et al. [22] designed and solved a neural ODE to complement the missing graph topology and unified spatial and temporal messaging, allowing for deeper graph propagation and fine-grained temporal message aggregation to characterize stable and accurate underlying spatial-temporal dynamics. Kong et al. [23] constructed a network to model relationships between stations, employing a deep clustering method based on graph neural networks to extract mobility patterns of bus stations. They designed a spatiotemporal prediction model named STGNNFormer based on Transformer to forecast bus station traffic, thereby enhancing prediction accuracy and efficiency.

Most of the aforementioned models overlook the dynamic spatial characteristics of traffic flow and only focus on extracting static spatial features. To address the issue of capturing the dynamic spatial characteristics in traffic flow, some dynamic evolution models based on GCN have appeared in recent years, such as Graph Attention Network (GAT) [24]. GAT calculates attention weights between nodes and their neighboring nodes, allowing each node to update its information as a weighted average of its neighbors. By doing so, GAT can more accurately capture the relationships between nodes and edges, thereby extracting richer information about dynamic spatial characteristics. Based on this, this paper proposes the MFSTBiSGAT deep learning framework, which aims to comprehensively describe the dynamic spatial characteristics of road networks by deeply mining the spatiotemporal features of traffic flow. The specific contributions are twofold:

1. Combining GAT with BiLSTM networks and utilizing the Spearman function to delve into hidden correlations among different nodes.
2. Employing three STBiSGAT components with identical structures to concurrently handle various traffic metrics, namely traffic flow, speed, and lane occupancy. This approach models spatiotemporal dependencies comprehensively, enabling a more thorough analysis of traffic flow conditions.

## Problem defintion

**Fig 1:**
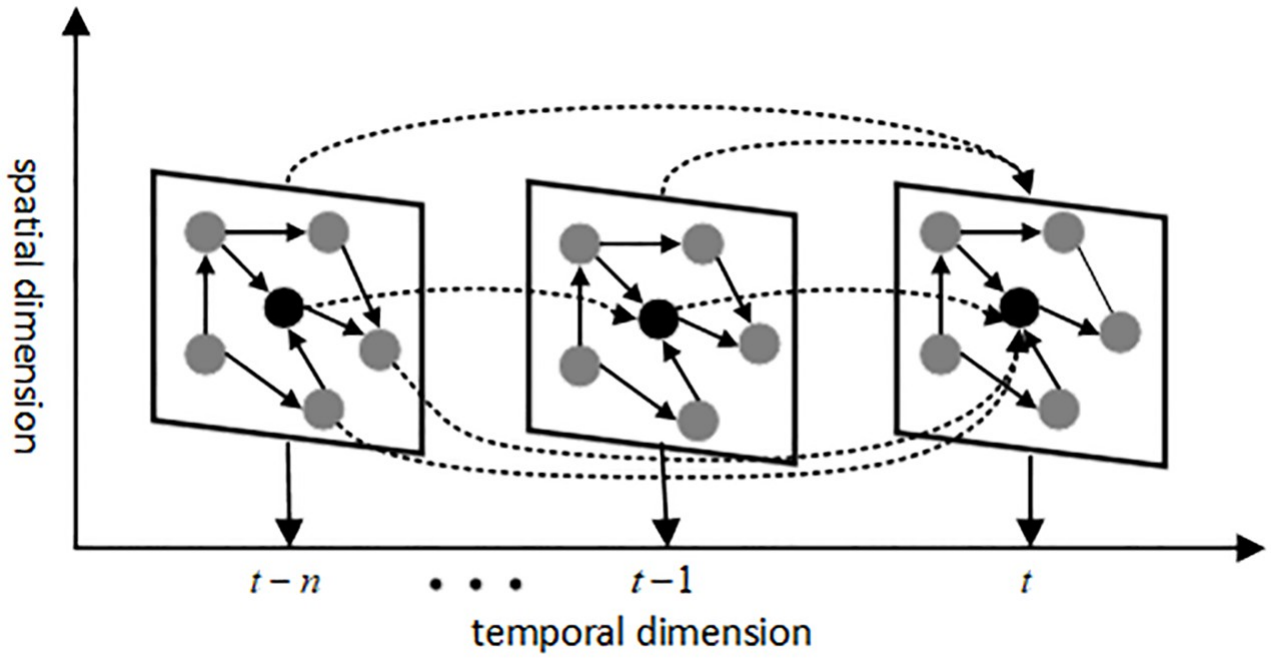
Spatio-temporal data.

This paper defines the traffic network as an undirected graph *G* = (*V*, *E*, *A*), each node in the graph represents a spatiotemporal graph data, as shown in Fig 1. Each time slice represents a spatial graph. As time progresses, it can be observed that the traffic flow of a given node is influenced not only by its historical data but also by the spatial information of adjacent nodes. In graph *G*, V = {v_1_, v_2_, ⋯, *v*_*N*_} is the set of nodes, where *N* is the number of nodes in the traffic network, *E* is the set of edges, and *A* is the adjacency matrix of graph *G*, *A* ∈ [0,1]^*N*×*N*^. Let the historical traffic flow sequence of a node be {*X*_*t*,*i*_|*t* = 1, 2, 3, …, *T*; *i* = 1, 2, 3, …, *N*}, where *X*_*t*,*i*_ represents the traffic flow data of node *i* at time *t*. Therefore, the short-term traffic flow prediction problem in the traffic network can be represented as Eq (1):

$$\left[X_{t+1},\cdots X_{t+T}\right]=f\left(G;\left(X_{t-n},\cdots X_{t-1},X_{t}\right)\right)$$ (1)

In the equation, *n* represents the length of the historical time series, *T* represents the length of the prediction time series, and *G* represents the representation of the distributed node topology structure relationship.

## Construction of traffic flow prediction model

**Fig 2:**
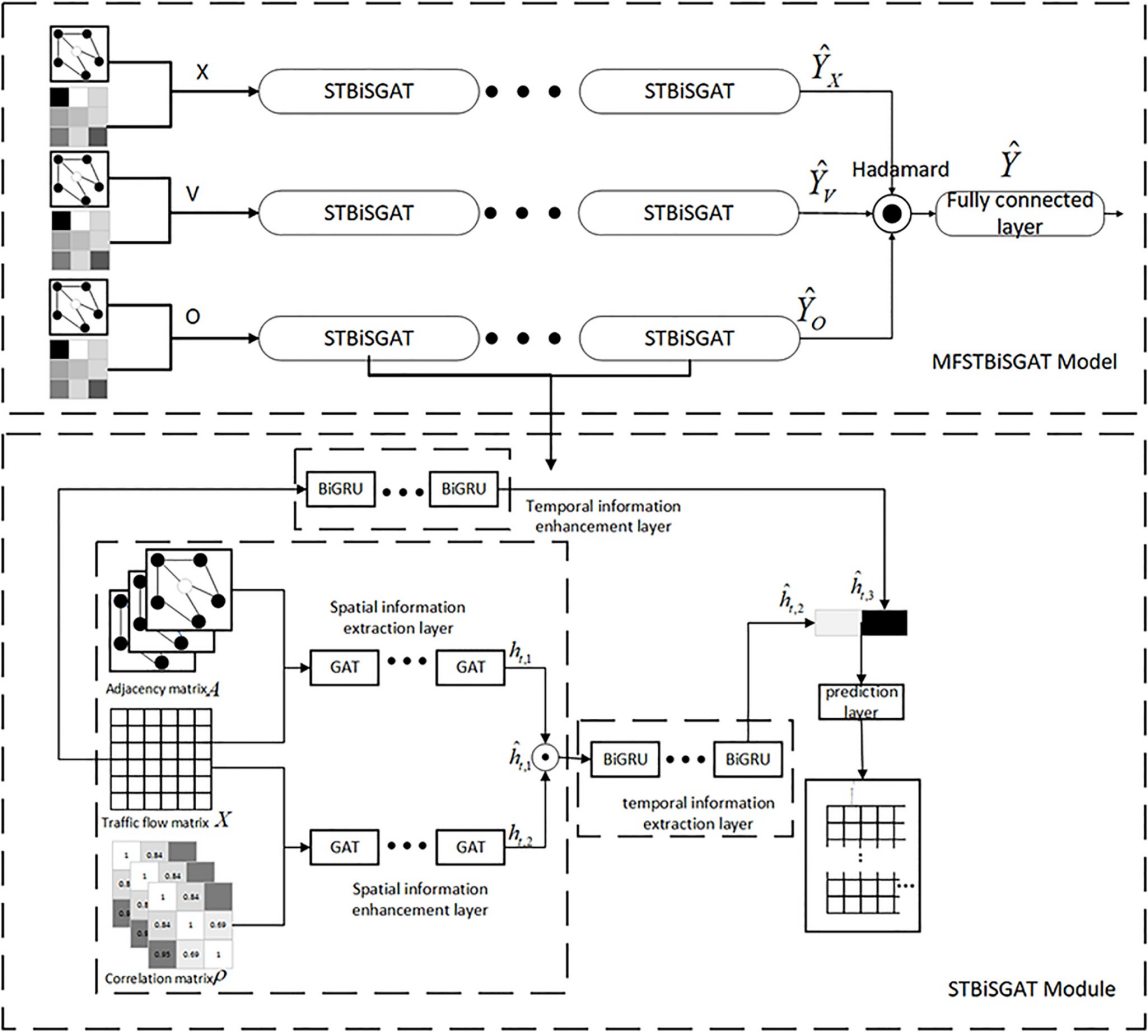
Overall architecture diagram.

To address the inadequacy of mining temporal and spatial information in traffic flow, this paper proposes the MFSTBiSGAT model, which enhances spatiotemporal information. The overall architecture of the model is shown in Fig 2. The model consists of three independent components, STBiSGAT, that have the same structure. Each component models the spatiotemporal dependencies of traffic flow, traffic speed and lane occupancy based on historical data. The STBiSGAT is composed of four main modules: spatial information extraction layer, temporal information extraction layer, temporal information enhancement layer and prediction layer. The first two layers are used to extract the spatial and temporal features of the road network, and the last two layers are used to obtain the temporal features of the original traffic flow information and to predict the future traffic flow based on the extracted spatial and temporal feature information.

### Spatial information extraction layer

The spatial information extraction layer consists of two parts: spatial feature extraction layer and spatial information enhancement layer. The spatial feature extraction layer considers the topological structure of the road network. The input is a matrix *X* formed by stacking the traffic flow time series of each node, and an adjacency matrix *A* generated from the input data in the distance file to represent the graph. By using GAT, the explicit spatial features *h*_*t*,1_ of the road network are extracted. The specific formula for aggregating the weighted contributions of node *j* to node *i* and obtaining the new features is as follows:

$$e_{ij}=a\left(x_{i},x_{j}\right)$$ (2)

$$\alpha_{ij}=\frac{\text{exp}\left(e_{ij}\right)}{\sum_{k\in N_{i}}\;\text{exp}\left({\text{e}}_{ik}\right)}$$ (3)

$$h_{i}^{\prime }=\sigma \left(\sum_{j\in N_{i}}\alpha_{ij}Wx_{j}\right)$$ (4)

where *x*_*i*_ and *x*_*j*_ are the feature vectors of node *i* and node *j* respectively. *e*_*ij*_ denotes the attention score between node *i* and node *j*, *a* corresponds to a single-layer feed-forward neural network used to compute the attention score between the two nodes, *α*_*ij*_ is the normalized attention weight, *W* is the corresponding learning parameter, and $h_{i}^{\prime }$ is the new feature representation of node *i*.

However, in order to enrich the expressiveness of the model, multi-head attention is introduced in GAT. The results obtained from *K* attention mechanisms are averaged to obtain the final new feature *h*_*i*_ of node *i*, as shown in Eq (5). Similarly, the explicit spatial feature *h*_*t*,1_ can be obtained by calculating the new features of all nodes.


$$h_{i}=\sigma \left(\frac{1}{K}\sum_{K=1}^{K}\sum_{j\in N_{i}}\alpha_{ij}^{K}W^{K}x_{j}\right)$$ (5)

The spatial information enhancement layer considers the complex nonlinear relationships among the nodes in the road network. It utilizes the Spearman correlation coefficient to uncover hidden correlations, which helps further enhance the spatial information.

**Fig 3:**
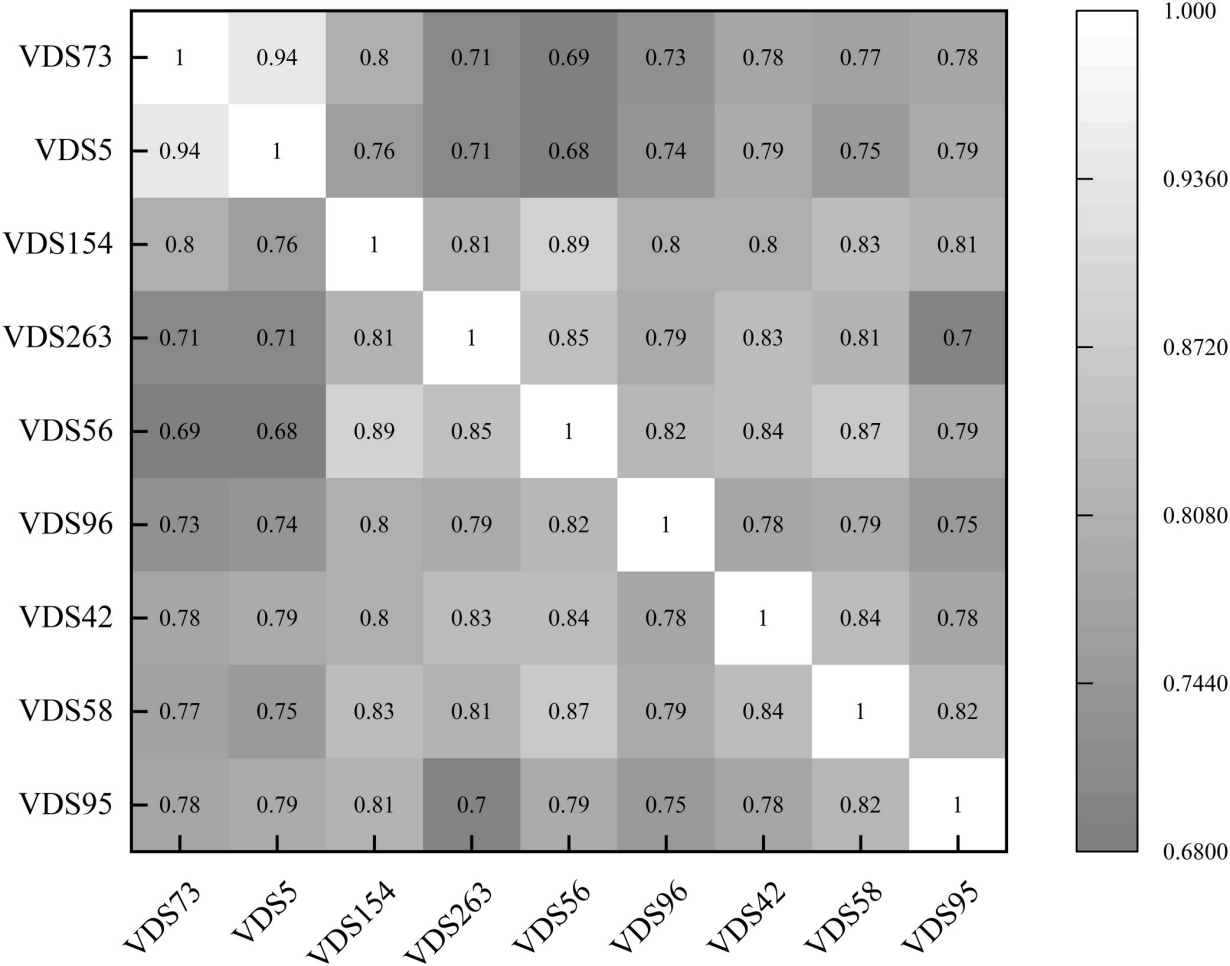
Heat map of node relationships.

As shown in Fig 3, the heat map of the relationship between nine neighboring nodes is generated using the Spearman correlation coefficient. The numerical values in the heatmap represent the degree of correlation for each corresponding node, with larger values indicating stronger correlations. Therefore, considering the implicit relationship between spatial nodes, the Spearman correlation coefficient is used here to extract the degree of correlation of the nodes and form the corresponding correlation coefficient matrix *ρ*, which is used to replace the adjacency matrix *A*, and is input to GAT with the traffic flow matrix *X*_*t*_ to obtain the implicit spatial features of the enhanced information *h*_*t*,2_. Its Spearman correlation coefficient is calculated as follows:

$$S=\frac{\frac{1}{n}\sum_{i=1}^{n}\left(R\left(x_{i}\right)-\bar{R\left(x\right)}\right)\cdot \left(R\left(y_{i}\right)-\bar{R\left(y\right)}\right)}{\sqrt{\left(\frac{1}{n}\sum_{i=1}^{n}{\left(R\left(x_{i}\right)-\bar{R\left(x\right)}\right)}^{2}\right)}\cdot \left(\frac{1}{n}\sum_{i=1}^{n}{\left(R\left(y_{i}\right)-\bar{R\left(y\right)}\right)}^{2}\right)}$$ (6)

Where *R*(*x*_*i*_) and *R*(*y*_*i*_) represent the bit of the ith data in the data of *x* node and *y* node, respectively, $\bar{R\left(x\right)}$ and $\bar{R\left(y\right)}$ denote the average bit in the data of *x* node and *y* node, respectively, where the bits are arranged according to the magnitude of the value of the traffic speed at each moment in time, and *n* denotes the total number of observed nodes.

The obtained explicit spatial features are fused with the implicit spatial features to obtain the final spatial features as follows:

$${\hat{h}}_{t,1}=\sigma (h_{t,1}⊙h_{t,2})$$ (7)

where *σ*(·) is the RELU activation function and ⊙ is the Hadamard product, which represents the multiplication of the corresponding elements of the matrix.

### Temporal information extraction layer

The dynamic nature of traffic flow data indicates that short-term traffic flows exhibit strong correlations, while traffic information exhibits long-term dependencies in terms of temporal features. The Bidirectional Long Short-Term Memory (BiLSTM) network can handle both forward and backward time series data, capturing longer-term dependencies and considering the dynamic features of data in previous and subsequent time periods. Therefore, BiLSTM is chosen in this paper to extract the temporal features of the traffic road network. The previously extracted spatial features ${\hat{h}}_{t,1}$ are used as input, and the forward hidden state ${\vec{h}}_{t,2}$ and backward hidden state ${\overset{\leftarrow }{h}}_{t,2}$ of the BiLSTM network are computed using Eqs (8) and (9), through the LSTM network. When both directions of the LSTM are trained, the outputs of the bi-directional hidden states are linked into new features ${\hat{h}}_{t,2}$ with spatio-temporal correlations via Eq (10) as follows:

$${\vec{h}}_{t,2}=\phi \left({\hat{h}}_{t,1}{\vec{w}}_{xh}+{\vec{h}}_{t-1}{\vec{w}}_{hh}+{\vec{b}}_{h}\right)$$ (8)

$${\overset{\leftarrow }{h}}_{t,2}=\phi \left({\hat{h}}_{t,1}{\overset{\leftarrow }{w}}_{xh}+{\overset{\leftarrow }{h}}_{t-1}{\overset{\leftarrow }{w}}_{hh}+{\overset{\leftarrow }{b}}_{h}\right)$$ (9)

$${\hat{h}}_{t,2}={\vec{h}}_{t,2}⊕{\overset{\leftarrow }{h}}_{t,2}$$ (10)

where ${\vec{w}}_{xh}$, ${\vec{w}}_{hh}$, ${\overset{\leftarrow }{w}}_{xh}$ and ${\overset{\leftarrow }{w}}_{hh}$ are the weight parameters of the model, ${\vec{b}}_{h}$ and ${\overset{\leftarrow }{b}}_{h}$ are the bias terms of the model, and *ϕ* is the tanh activation function.

### Temporal information enhancement layer

In order to prevent each highway from overly relying on surrounding road information while maintaining its own personalized time series features, inspired by the ResNet network structure, BiLSTM is specifically used to extract the temporal features of the original input data, enriching the temporal information of traffic flow. The specific process is similar to the previous time feature extraction, but here the original traffic flow matrix *X*_*t*_ is used as input, and the enhanced temporal features ${\hat{h}}_{t,3}$ are obtained through the BiLSTM network. The specific formula is as follows:

$${\vec{h}}_{t,3}=LSTM\left(X_{t},{\vec{h}}_{t-1}\right)$$ (11)

$${\overset{\leftarrow }{h}}_{t,3}=LSTM\left(X_{t},{\overset{\leftarrow }{h}}_{t-1}\right)$$ (12)

$${\hat{h}}_{t,3}={\vec{h}}_{t,3}⊕{\overset{\leftarrow }{h}}_{t,3}$$ (13)

### Prediction layer

The prediction layer of this model consists of a fully connected network. It concatenates the spatiotemporal features ${\hat{h}}_{t,2}$ extracted from the previous layers with the temporal features ${\hat{h}}_{t,3}$ extracted from the temporal information enhancement layer, resulting in the feature ${\hat{h}}_{t}$. The fully connected network assigns different weights to ${\hat{h}}_{t}$ and finally outputs the predicted traffic flow $\bar{Y}$. The specific formula is as follows:

$${\hat{h}}_{t}={\hat{h}}_{t,2}⊕{\hat{h}}_{t,3}$$ (14)

$$\bar{Y}=w{\hat{h}}_{t}+b$$ (15)

where *w* is the weight parameter and *b* is the bias term.

### Prediction results

Under the same road conditions, when the traffic flow increases, the mutual influence and competition among vehicles increase, leading to vehicle deceleration and a decrease in traffic speed. Thus, there is a negative correlation between traffic flow and traffic speed. At the same time, as traffic volume increases, the proportion of occupied lanes also increases. When traffic volume is low, there is a relatively large gap between vehicles, resulting in a lower lane occupancy rate. Thus, the two factors are positively correlated. From this, it can be seen that there is a close relationship between traffic volume, traffic speed and lane occupancy rate. Incorporating traffic speed and lane occupancy rate into the model helps improve the model’s robustness. Specifically, the MFSTBiSGAT model utilizes the STBiSGAT module to separately predict future traffic volume ${\hat{Y}}_{x}$, traffic speed ${\hat{Y}}_{v}$ and lane occupancy rate ${\hat{Y}}_{o}$. It then learns the fusion impact of different components from historical traffic data, as shown in Eq (16).

$$\hat{Y}=\sigma ({\hat{Y}}_{x}⊙{\hat{Y}}_{v}⊙{\hat{Y}}_{o})$$ (16)

where $\hat{Y}$ is the predicted traffic flow after fusing the features, *σ*(·) is the *relu*(·) activation function, and ⊙ is the Hadamard product, which represents the multiplication of the corresponding elements of the matrix.

## Experimental results and analysis

### Experimental dataset

This article selected two real-world datasets, PeMSD4 and PeMSD8, as experimental data. Both datasets were collected from the Performance Measurement System (PeMS) in California. The data in these datasets are aggregated every 5 minutes. The PeMSD4 dataset contains traffic state data from road network nodes in the San Francisco Bay Area, California, while the PeMSD8 dataset contains traffic state data from road network nodes in San Bernardino County, California, USA. By conducting comparative experiments on these two datasets, this article aims to validate the effectiveness of the improved model. Detailed information is shown in Table 1.

**Table 1:** The information of the PeMS dataset used.

Dataset	Number of nodes	Length	Time span
PeMSD4	307	16992	2018/1/1-2018/2/28
PeMSD8	170	17856	2016/7/1-2016/8/31

When there are missing data points in the dataset, this article uses linear interpolation to recover the missing data. Additionally, the data is standardized using the Z-Score method. Finally, the dataset is divided into two parts: a training set and a test set, with a ratio of 8:2.

### Evaluation metrics

This article uses Mean Absolute Error (MAE) and Root Mean Square Error (RMSE) as performance metrics in the experiments. Their mathematical expressions are as follows:

$$MAE=\frac{1}{k}\overset{k}{\underset{i=1}{\sum }}\left|{\hat{y}}_{i}-y_{i}\right|$$ (17)

$$RMSE=\sqrt{\frac{1}{k}\overset{k}{\underset{i}{\sum }}{\left({\hat{y}}_{i}-y_{i}\right)}^{2}}$$ (18)

where ${\hat{y}}_{i}$ denotes the predicted value, *y*_*i*_ denotes the true value and *k* denotes the number of samples.

### Experimental settings

All experiments were conducted using a Win10 system with hardware configuration including an NVIDIA GeForce RTX 2080Ti GPU and 13th Gen Intel(R) Core^™^ i5-13400. The prediction task of the MFSTBiSGAT model was implemented based on the PyTorch deep learning framework. In the model presented in this article, a sliding window approach is used with a window size of 12 historical time steps, equivalent to 1 hour, to predict the future 1 hour of traffic volume. After many experiments, to achieve better results, this article uses the Adam optimizer. The initial learning rate is set to 1e-3. The batch size for training samples is set to 64. The number of iterations is set to 60. The BiLSTM has 4 layers, with 64 neurons in the input layer and 64 neurons in the hidden layer. To prevent overfitting and improve model generalization, a dropout rate of 0.3 is set.

### Hyperparameter experiments

**Fig 4:**
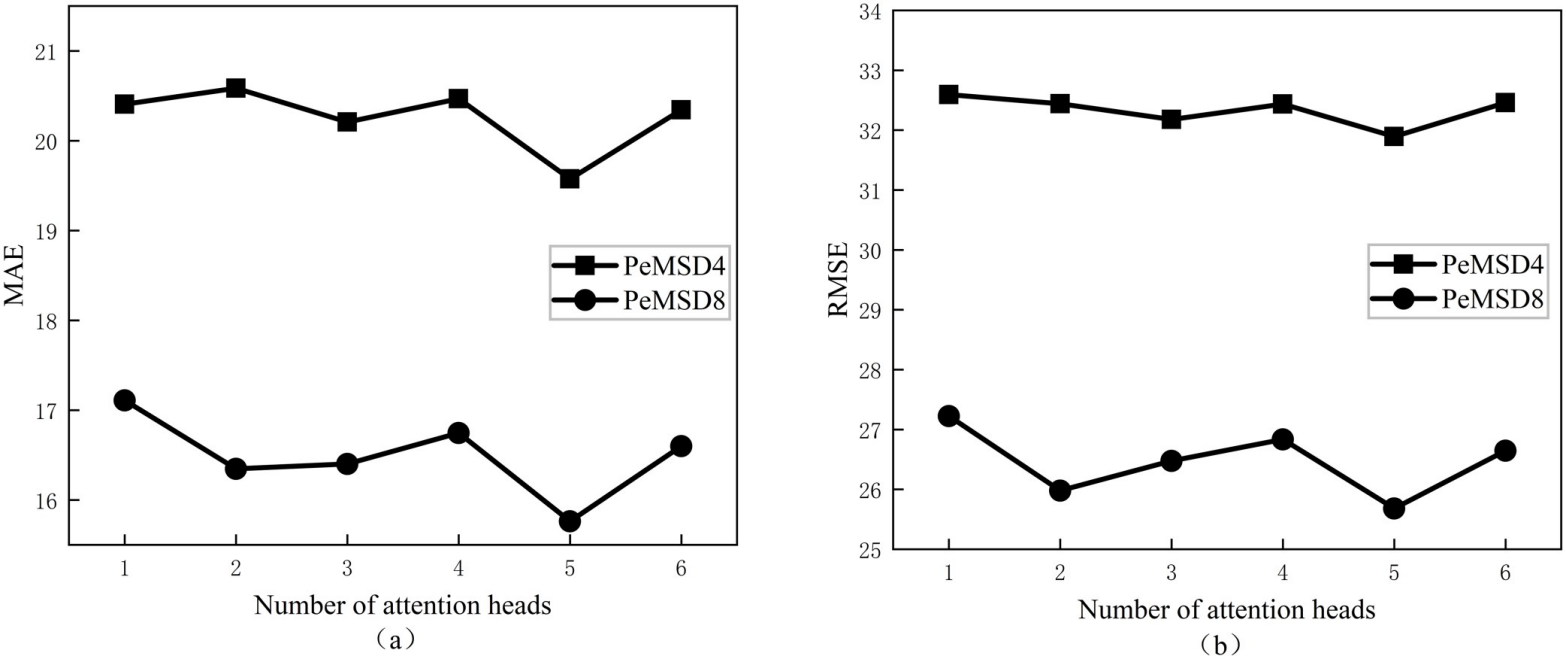
Effect of attentional head count on evaluation indicators. (a)The effect of the number of attention heads on MAE. (b)The effect of the number of attention heads on RMSE.

Increasing the number of attention heads K in multi-head attention can improve the performance of the model. However, having too many attention heads can lead to overfitting or excessive computation. Therefore, it is necessary to experiment and optimize the number of attention heads based on the specific task and dataset to find the optimal balance. In order to find the optimal number of attention heads K, this article conducted six sets of comparative experiments with K as the only variable, and the results are shown in Fig 4. It is evident from the figure that the MFSTBiSGAT model achieves the lowest MAE and RMSE values when K = 5 on both datasets. This suggests that the model can effectively extract features from multiple subspaces, thereby enhancing its expressive power. Therefore, in order to improve the accuracy and expressive power of the model, the attention head number K of GAT is set to 5, and the hidden layer dimension is set to 32.

### Experimental results and analysis

In the experiments, this article conducted performance validation on the PeMSD4 and PeMSD8 datasets. According to the results shown in Table 2, the MFSTBiSGAT model performs the best on both datasets. In comparison, models such as HA, BPNN and LSTM that only consider temporal features perform poorly because they overlook the spatial characteristics of the road network. Similarly, the GCN model that only considers spatial features also has certain limitations. Therefore, combining temporal and spatial features is essential for accurate traffic flow prediction. In this regard, the GCN-BiLSTM model successfully integrates the GCN network into the BiLSTM architecture, effectively capturing the complex spatial dependencies between road segments and the temporal patterns of traffic flow. As a result, it improves the prediction accuracy.

**Table 2:** Comparison of performance metrics for different models.

Models	PeMSD4		PeMSD8	
	MAE	RMSE	MAE	RMSE
HA [4]	38.24	56.68	31.92	47.42
BPNN [9]	36.31	51.31	31.25	44.06
GCN [15]	28.00	41.78	22.14	33.80
LSTM [17]	25.82	39.12	21.43	32.25
CNN-TCN [25]	25.12	39.17	20.40	29.89
GCN-BiLSTM [26]	24.73	38.05	20.22	30.63
MSTGCN [21]	24.99	38.04	19.55	29.58
GAT-GRU [27]	23.44	37.25	19.05	29.28
ASTGCN [21]	21.98	34.57	18.03	27.74
STGODE [22]	21.20	32.96	16.59	25.89
Dstagnn [28]	**19.42**	31.95	15.96	25.41
MFSTBiSGAT	19.57	**31.89**	**15.76**	**25.68**

**Fig 5:**
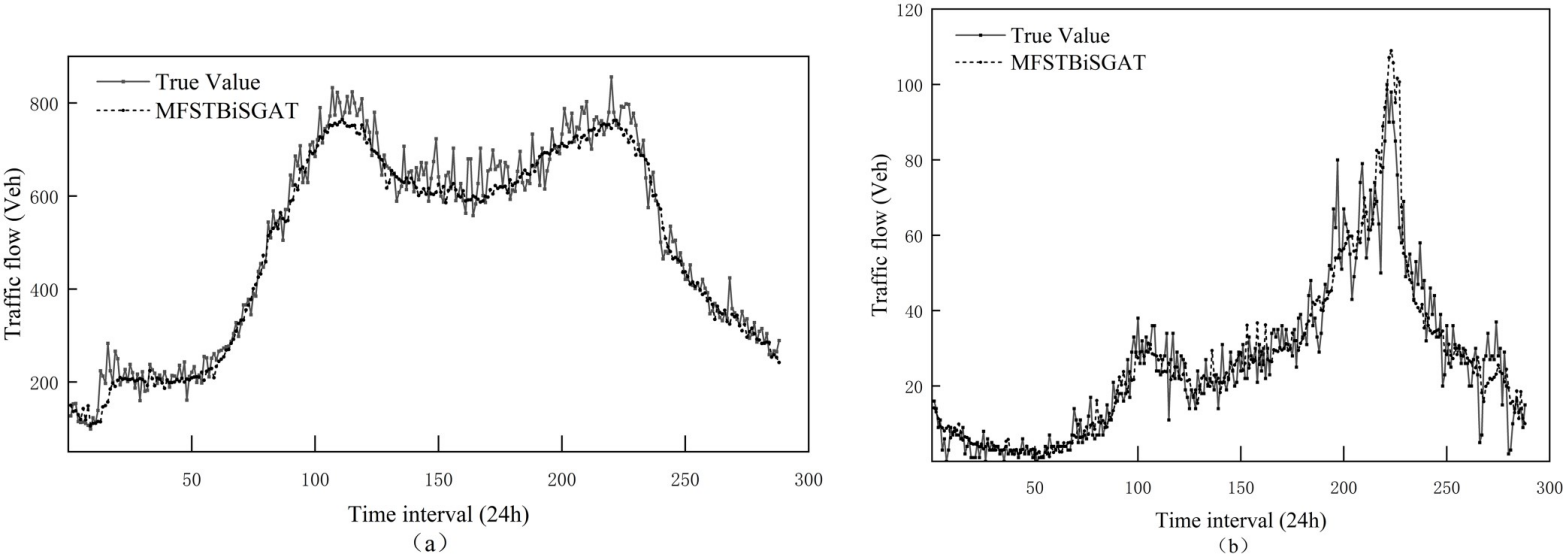
MFSTBiSGAT predicts performance of results. (a)MFSTBiSGAT is on node 101 of the 04 dataset. (b)MFSTBiSGAT is on node 108 of the 08 dataset.

However, the MFSTBiSGAT model performs better in terms of prediction accuracy, and the specific experimental results are shown in Fig 5. Compared to the GCN-BiLSTM model, on the PeMSD4 dataset, MFSTBiSGAT resulted in a 20.86% and 16.19% reduction in MAE and RMSE, respectively. In addition, MFSTBiSGAT outperforms another model, MSTGCN, which considers both temporal and spatial features, resulting in 21.69% and 16.17% reduction in MAE and RMSE, respectively. Compared to GAT-GRU, the proposed STBiSGAT model reduces the MAE and RMSE by 16.51% and 14.39% respectively. Compared to ASTGCN, the proposed STBiSGAT model reduces the MAE and RMSE by 10.96% and 7.75% respectively. Compared to STGODE, MFSTBiSGAT reduces the MAE and RMSE by 7.69% and 3.25% respectively. Compared to the DSTAGNN model, only one metric is larger, while the other three metrics are lower. It can be seen that the MFSTBiSGAT method in this paper fully considers the spatio-temporal dynamic features, effectively captures the evolutionary trends of nodes, and thus describes the behavior of nodes in the spatio-temporal domain.

### Ablation analysis

To further investigate the superiority of our model in this study, we conducted ablation experiments for comparison. Three variants based on the MFSTBiSGAT model are designed and compared to predict the change in traffic flow error for different future time steps (5min-60min), the details of which are given below.

a. MFSTBiSGAT-S: The spatial information enhancement layer has been removed.
b. MFSTBiSGAT-T: The temporal information enhancement layer has been removed.
c. MFSTBiSGAT-MF: The features of traffic speed and lane occupancy have been removed.

**Fig 6:**
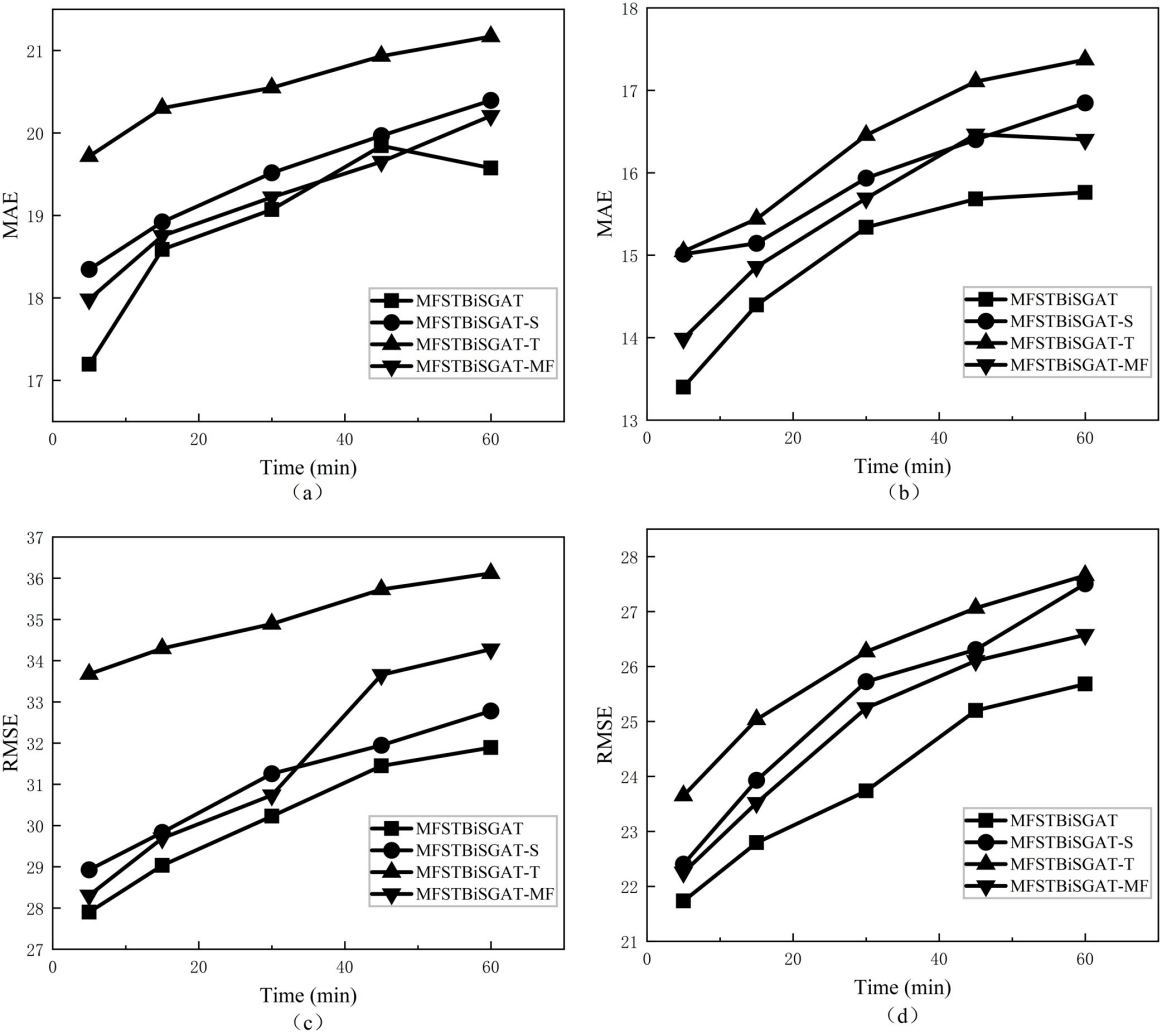
Prediction errors for each model at different time steps on the two datasets. The MAE and RMSE errors on the PeMSD4 dataset are denoted as (a) and (b), respectively. The MAE and RMSE errors on the PeMSD8 dataset are denoted as (c) and (d), respectively.

As shown in Fig 6, based on the results of the evaluation metrics in the figure, the MFSTBiSGAT model exhibits the best predictive performance. It can be observed that the spatial information enhancement layer, temporal information enhancement layer, and other features are all important for the predictions of this model. Due to the complex nonlinear relationships between nodes in spatial domain, the MFSTBiSGAT-S model, which removes the module for capturing these hidden correlations, exhibits relatively lower performance. Traffic flow exhibits significant temporal dynamics, and the MFSTBiSGAT-T model, due to the loss of some temporal features during the early extraction of spatial features, is unable to accurately capture the original temporal characteristics of the raw traffic flow. As a result, the model exhibits the poorest predictive performance. The MFSTBiSGAT-MF model, due to the removal of other features, exhibits reduced robustness, leading to a slight decrease in overall predictive performance. From the trends of the evaluation metrics in the figure, it is evident that as the prediction horizon increases, the errors of all models gradually increase.

## Conclusion

In response to the issue of existing models not effectively exploring the spatiotemporal features of traffic flow, we propose the MFSTBiSGAT model for traffic flow prediction based on graph deep learning framework. This model combines the advantages of GAT and BiLSTM. It can extract the spatial dynamic features of the traffic network and capture the temporal evolution trends of traffic flow from both the past and future directions. In addition, to explore spatio-temporal features in depth, the model captures hidden correlations between nodes in spatial feature extraction, extracts the original temporal features of the traffic network in the temporal dimension, and incorporates closely related features such as traffic speed and lane occupancy, which are closely related to traffic flow, through Hadamard product and concatenation operations. This effectively enhances the expressive power of spatiotemporal information in the model.

After conducting numerous experiments on two real-world datasets, the MFSTBiSGAT model has demonstrated significantly better predictive accuracy compared to other baseline models. Compared to recent spatiotemporal prediction models, MFSTBiSGAT achieves a reduction in MAE values ranging from 7.69% to 21.69% and a reduction in RMSE values ranging from 3.25% to 16.19% on the PeMSD4 dataset. Results from ablation experiments show that the MFSTBiSGAT model outperforms models without the spatiotemporal information enhancement layer and multiple features, further confirming the effectiveness of the added modules.

However, there are some of the limits of our study. Future traffic flow prediction models will also consider additional factors such as weather conditions, holidays, and other relevant factors. These factors have a significant impact on traffic conditions and often exhibit sudden or periodic patterns. Therefore, incorporating these factors into the model is meaningful for improving the accuracy of predictions.
